# New *Resinogalea* species from *Araucaria araucana* resin in Chile and reclassification of the genus in the *Cryptocaliciomycetidae*

**DOI:** 10.1186/s43008-023-00122-9

**Published:** 2023-08-18

**Authors:** Felipe Balocchi, Irene Barnes, Michael J. Wingfield, Rodrigo Ahumada, Cobus M. Visagie

**Affiliations:** 1 Department of Plant and Soil Sciences, Forestry and Agricultural Biotechnology Institute (FABI), University of Pretoria, Pretoria, 0002 South Africa; 2 Department of Biochemistry, Genetics and Microbiology, FABI, University of Pretoria, Pretoria, 0002 South Africa; 3 Silviculture and Forest Health Division, Bioforest, Concepción, Chile

**Keywords:** Two new taxa, Monkey puzzle tree, Resinicolous fungi, *Resinogalea araucana*, *Resinogalea tapulicola*

## Abstract

*Araucaria araucana* is an ancient conifer, native to the mountain ranges in Chile and Argentina. These trees host a large number of organisms, mainly insects, strongly or even exclusively associated with them. The recent emergence of a novel canker disease on *A. araucana* has emphasised the importance of fungi associated with these iconic trees and has resulted in the discovery of various new species. In this study, we considered the identity of an unknown calicioid fungus consistently found on resin on the branches of *A. araucana*. Preliminary phylogenetic analyses placed isolates in the recently described sub-class *Cryptocaliciomycetidae*, closest to *Cryptocalicium blascoi*. However, the morphology of the ascomata and its occurrence in a unique niche suggested that the closest relative could be *Resinogalea humboldtensis* (*Bruceomycetaceae*, *incertae sedis*), a fungus with similar sporing structures found on resin of *Araucaria humboldtensis* in New Caledonia. There are no living cultures or sequence data available for either *R. humboldtensis* or its supposed closest relative, *Bruceomyces castoris,* precluding sequence-based comparisons. Morphological comparisons of the sporing structures on *A. araucana* confirmed that the ascomatal morphology of our unknown calicioid fungus and *R. humboldtensis* are almost identical and resemble each other more so than *B. castoris* or *Cr. blascoi*. A phylogenetic analysis based on the small subunit (SSU), internal transcribed spacer (ITS) and large subunit (LSU) rDNA regions resolved our strains into two clades with *Cr. blascoi* as its closest relative. Further analyses applying the Genealogical Concordance Phylogenetic Species Recognition (GCPSR) based on ITS, mini chromosome maintenance protein complex (*MCM7*), RNA polymerase II second largest subunit (*RPB2*) and translation elongation factor 1-alpha (*TEF*) gene regions, confirmed that strains represent two new species. Based on our morphological observations and phylogenetic analyses, we introduce two new *Resinogalea* species, *R. araucana* and *R. tapulicola,* and reclassify the genus in the subclass *Cryptocaliciomycetidae*.

**Supplementary information:**

The online version contains supplementary material available at 10.1186/s43008-023-00122-9.

## INTRODUCTION

*Araucaria araucana* (*Araucariaceae*), commonly known as Araucaria, pewén or the monkey puzzle tree, is an ancient species native to the mountain ranges of Chile and Argentina. The species is one of only two in the genus and family found in South America, with the remainder distributed in the Southeast Asia–Oceania region (Balocchi et al. 2022b). These trees are associated with a diverse range of organisms, thought to have co-evolved with them and that are considered scientifically rare (Kuschel 2000). Insects on these trees have been well studied with several species strictly associated with *Araucaria* (Kuschel 2000; Carpintero and Dellapé 2006; Beéche 2017). In contrast, very little is known regarding the microbiology of these iconic trees; a situation that also extends to most members of the *Araucariaceae* (Balocchi et al. 2022b). A newly discovered canker disease on *A. araucana* in Chile (Balocchi et al. 2021) drew attention to microfungi on these trees. The new genus *Pewenomyces* and four new species were subsequently described in the *Coryneliaceae* with *P. kutranfy* shown to be the causal pathogen (Balocchi et al. 2022a). During studies to resolve the identity of the canker disease, ascomata of a rarely occurring calicioid (i.e. stipitate, capitate and mazaediate) fungus were observed emerging from dry resin patches associated with cankers or other sources of wounding such as insect feeding or mechanical damage.

Based on morphological features and niche preference, the unusual ascomata were considered to be most similar to the recently described *Resinogalea humboldtensis* (*Bruceomycetaceae,* i*ncertae sedis*) (Rikkinen et al. 2016). *Resinogalea humboldtensis* was described from resin patches arising from insect damage on *Araucaria humboldtensis* in New Caledonia. This species is known only from its ascomata, with no live cultures or DNA sequences available. Based on morphology, Rikkinen et al. (2016) considered that *R. humboldtensis* did not resemble other previously known non-lichenized resinicolous calicioid fungi, which reside mostly in the *Mycocaliciomycetidae*. Rather, it was more similar with *Bruceomyces castoris* (syn. *Brucea castoris*) (*incertae sedis*, *Ascomycota*) (Rikkinen 2003), a fungus found on resin resulting from beaver feeding on North American conifers. This resulted in the description of the *Bruceomycetaceae* (*incertae sedis*, *Ascomycota*) to accommodate both genera, *Resinogalea* and *Bruceomyces*, each containing a single species (Rikkinen et al. 2016). However, the lack of DNA sequences and living cultures for these two species leaves the Family unresolved in terms of higher classification.

Recently, Prieto et al. (2021) described the subclass *Cryptocaliciomycetidae,* a new lineage of non-lichenized calicioid fungi in the *Eurotiomycetes* based on *Cryptocalicium blascoi*, a new species found on bark and resin of *Juniperus* and *Cupressus* species in Spain. This novel fungus resembled the fungus we found in *A. araucana* and had DNA sequences and living cultures available for comparisons. Consequently, the aim of the present study was to consider the identity of our fungus on *A. araucana*, using both morphological characterisation and phylogenetic inference and furthermore, to identify its closest relatives.

## METHODS

### Sample collections and fungal isolations

*Araucaria araucana* samples were collected from different areas in Chile where these trees occur naturally (Balocchi et al. 2021). These included the Conguillío National Park (sector Los Paraguas), Villarrica National Park (sector Puesco), Ralco Natural Reserve and private areas on the Coastal range (Nahuelbuta, Trongol Alto). Sample collections were carried out between 2017 and 2020 during the Chilean summer periods (December–February). Samples consisted mainly of branch segments and a few stems from natural regeneration that exhibited varied sources of damage due to fungal cankers or insect feeding, leading to the production of resin.

Isolations from plant material were made by first surface disinfesting the tissues by immersion in 70% ethanol for 10 s, followed by 1 min in 5% sodium hypochlorite, and rinsed in sterile distilled water. Tissue specimens were then plated on ½ strength potato dextrose agar (½PDA: 19.5 g/L PDA, 10 g/L agar; Merck) or 2% malt extract agar (MEA: 20 g/L malt extract, 20 g/L agar; Biolab) in Petri dishes. Plates were incubated for 14 d at 20–25 °C. Fungal colonies of interest were transferred to new MEA plates in order to produce pure cultures. Isolates were further purified by transferring them to 2% water-agar (WA: agar 20 g/L; Biolab) and allowing these to grow for 2–4 d before transferring single hyphal tips to new MEA plates. Direct isolations from ascomata were made by collecting ascospores with a sterile needle, spreading these onto WA and after 2–4 days, transferring germinating single spores to new MEA plates.

All relevant isolates used in this study were deposited into the culture collection (CMW) housed at the Forestry and Agricultural Biotechnology Institute (FABI: University of Pretoria, Pretoria, South Africa). Representative strains for each species were subsequently deposited into the CMW-IA culture collection (Innovation Africa: University of Pretoria, Pretoria, South Africa) and to the culture collection (CBS) of the Westerdijk Fungal Biodiversity Institute (Utrecht, The Netherlands). Specimens were deposited in the PREM fungarium of the South African National Collections of Fungi housed at the Agricultural Research Council (ARC: Plant Health and Protection, Roodeplaat, South Africa).

### DNA extractions, PCR, and sequencing

DNA extractions were performed using the Prepman® Ultra Sample Preparation Reagent kit (Thermo Fisher Scientifc, Waltham, MA) following the manufacturer’s protocols. For some strains, DNA was extracted using a phenol–chloroform method. For the latter approach, strains were grown on MEA for 21 d, after which mycelium was collected in 2 mL Eppendorf tubes and freeze dried overnight. Mycelia was ground by adding metal beads and shaking this in a mixer mill (30 oscillations/s for 3 min; MM 301, Retsch) and then following the protocol described by Barnes et al. (2001) and modified by Balocchi et al. (2021). The quality and quantity of DNA for each extraction was measured using a NanoDrop™ spectrophotometer (Thermo Scientific NanoDrop ND-1000), with working stocks subsequently standardised to ~ 30 ng/µL for each sample.

Six gene regions were amplified through PCR, which included: (i) the internal transcribed spacer 5.8S rDNA (ITS) with primers ITS1 & ITS4 (White et al. 1990); (ii) the nuclear 28S large ribosomal subunit (LSU) with primers LROR & LR5 (Vilgalys and Hester 1990; Rehner and Samuels 1995); (iii) the nuclear 18S small ribosomal subunit (SSU) with primer pairs NS1 & NS4 and NS3 & NS8 (White et al. 1990); (iv) the RNA polymerase II second largest subunit (*RPB2*) with primers RPB2-5f2 & RPB2-7cR (Liu et al. 1999; Sung et al. 2007); (v) the mini chromosome maintenance protein complex 7 (*MCM7*) with primers Cer-MCM7F & Cer-MCM7R (De Beer et al. 2014); and (vi) the translation elongation factor 1-alpha (*TEF1*) large intron with primers EF1-782F & EF-2 (O’Donnell et al. 1998; Carbone and Kohn 1999). PCRs were prepared in 25 µL reaction volumes and contained 5 µL 5X MyTaq™ Reaction Buffer (Bioline, London, UK), 0.5 µL (1 µL for *MCM7*) of each primer (10 µM), 0.3 µL MyTaq™ DNA Polymerase and 17.7 µL (16.7 µL for *MCM7*) sterile deionized water. The thermal cycling conditions included an initial denaturation step of 95 ˚C for 3 min, followed by 35 cycles of denaturing at 95 ˚C for 30 s, annealing at 56 ˚C (52 ˚C for *TEF1* and *MCM7*) for 30 s and elongation at 72 ˚C for 45 s, followed by a final elongation step at 72 ˚C for 4 min. Successful amplifications were confirmed by staining PCR amplicons with GelRed® (2 µL per 4 µL of PCR product) and electrophoresing the product on a 1% agarose gel for 12 min at 110 V.

PCR amplicons were cleaned from excess primers and unincorporated nucleotides either by sodium acetate precipitation (Duong et al. 2013) or using ExoSAP-IT™ PCR Product Cleanup Reagent (Applied Biosystems™, Thermo Fisher) following the manufacturer's instructions. Amplicons were sequenced in both directions using the BigDye® Terminator Cycle Sequencing Kit (Applied Biosystems™, Thermo Fisher) following the manufacturer's instructions. Sanger sequencing was carried out at the DNA Sanger sequencing facility of the Faculty of Natural and Agricultural Sciences, University of Pretoria. The obtained sequences were assembled into consensus sequences using CLC Main Workbench v.21.0.3 (Qiagen, Hilden, Germany). Newly generated sequences (Table 1) were deposited into GenBank (www.ncbi.nlm.nih.gov/genbank/).

**Table 1:** Taxa used for phylogenetic analyses, including representatives of the *Eurotiomycetes* and subsequent ranks, and *Lecanoromycetes* used as outgroup

Taxonomic position		Isolate	ITS	LSU	SSU	*RPB2*	*MCM7*	*TEF*
*EUROTIOMYCETES*								
*Chaetothyriomycetidae*								
*Chaetothyriales*	*Cyphellophora guyanensis*	MUCL 43737	NR_132880	KC455253	NG_065005	–	–	–
	*Exophiala eucalyptorum*	CBS 121638	KC455245	KC455258	KC455302	–	–	–
	*Knufia perforans*	CBS 885.95	NR_121502	NG_042586	NG_062148	–	–	–
	*Vonarxia vagans*	CBS 123533	FJ839636	FJ839672	KC455310	–	–	–
*Pyrenulales*	*Pyrenula nitida*	F-5929	JQ927458	DQ329023	–	–	–	–
	*Pyrgillus javanicus*	AFTOL-ID 342	DQ826741	DQ823103	DQ823110	–	–	–
	*Rhynchostoma proteae*	CBS 112051	NR_132824	NG_073789	–	–	–	–
*Verrucariales*	*Catapyrenium cinereum*	AFTOL-ID 2230	–	EF643747	EF689829	–	–	–
	*Verrucaria lecideoides*	AFTOL-ID 2295	EU010256	EF643798	–	–	–	–
	*Verrucaria muralis*	AFTOL-ID 2265	EU010261	EF643803	EF689878	–	–	–
*Coryneliomycetidae*								
*Coryneliales*	*Caliciopsis pinea*	CBS 139.64	KP881691	DQ678097	DQ678043	EF411067	**OP524467**	OM982873
	*Caliciopsis pseudotsugae*	CBS 140.64	MT334518	MT334517	MT359911	–	–	–
	*Corynelia africana*	PREM 57242	NR_153901	NG_058910	KP881719	–	–	–
	*Corynelia fructigena*	PREM 57240	NR_153902	NG_058911	KP881720	–	–	–
	*Lagenulopsis bispora*	PREM 57232	NR_154120	NG_060325	NG_061200	–	–	–
	*Pewenomyces kutranfy*	CMW 54240	NR_172182	MT334515	NG_074914	–	–	–
*Cryptocaliciomycetidae *								
*Cryptocaliciales*	*Cryptocalicium blascoi*	ARAN-Fungi 14,723	MW999967	MW999967	–	MZ020967	MZ020966	–
		Etayo 31,798	MW999969	MW999951	MW999950	–	–	–
	***Resinogalea araucana*** sp. nov (sp. 1)	CMW 57581	**OP508124**	**OP508136**	**OP508112**	**OP524474**	**OP524460**	**OP524486**
		CMW 53536	**OP508119**	**OP508131**	**OP508107**	**OP524469**	**OP524455**	**OP524481**
		CMW 53539	**OP508120**	**OP508133**	**OP508108**	**OP524470**	**OP524456**	**OP524482**
		CMW 53543	**OP508121**	**OP508134**	**OP508110**	**OP524471**	**OP524457**	**OP524483**
		CMW 53544	**OP508122**	**OP508132**	**OP508109**	**OP524472**	**OP524458**	**OP524484**
		CMW 57580	**OP508123**	**OP508135**	**OP508111**	**OP524473**	**OP524459**	**OP524485**
	***Resinogalea tapulicola*** sp. nov (sp. 2)	CMW 53537	**OP508126**	**OP508138**	**OP508114**	**OP524476**	**OP524462**	**OP524488**
		CMW 53535	**OP508125**	**OP508137**	**OP508113**	**OP524475**	**OP524461**	**OP524487**
		CMW 53538	**OP508127**	**OP508139**	**OP508115**	**OP524477**	**OP524463**	**OP524489**
		CMW 53540	**OP508128**	**OP508140**	**OP508116**	**OP524478**	**OP524464**	**OP524490**
		CMW 53542	**OP508129**	**OP508141**	**OP508117**	**OP524479**	**OP524465**	**OP524491**
		CMW 57582	**OP508130**	**OP508142**	**OP508118**	**OP524480**	**OP524466**	**OP524492**
*Eurotiomycetidae*								
*Arachnomycetales*	*Arachnomyces minimus*	CBS 324.70	–	NG_056963	AJ315167	–	–	–
	*Arachnomyces nodosetosus*	CCF 3975	HM205102	HM205103	HM205104	–	–	–
	*Arachnomyces peruvianus*	CBS 112.54	NR_160079	NG_057623	–	–	–	–
*Euriotiales*	*Aspergillus cremeus*	NRRL 5081	NR_137455	NG_057314	NG_063231	–	–	–
	*Aspergillus herbariorum*	DAOM 221134	JN942870	JN938918	JN938995	–	–	–
	*Aspergillus penicillioides*	NRRL 4548	NR_131285	NG_057322	NG_063229	–	–	–
	*Byssochlamys nivea*	CBS 100.11	NR_144910	NG_058631	NG_061072	–	–	–
	*Penicillium expansum*	DAOM 215345	JN942855	JN938952	JN938958	–	–	–
	*Penicillium taxi*	CBS 206.57	NR_145184	NG_057624	NG_062613	–	–	–
	*Sagenomella diversispora*	CBS 354.36	NR_164428	NG_063981	NG_060979	–	–	–
	*Sagenomella verticillata*	CBS 414.78	NR_160158	NG_064113	NG_062610	–	–	–
	*Thermoascus crustaceus*	CBS 181.67	NR_144915	NG_064060	NG_062804	–	–	–
	*Thermoascus verrucosus*	CBS 605.74	NR_103601	NG_075362	NG_074947	–	–	–
	*Trichocoma paradoxa*	CBS 788.83	JN899398	FJ358290	FJ358354	–	–	–
*Onygenales*	*Amauroascus verrucosus*	NFCCI 2672	NR_160558	JQ517293	JQ517294	–	–	–
	*Aphanoascus verrucosus*	NBRC 32381	NR_131309	NG_057011	–	–	–	–
	*Apinisia graminicola*	CBS 721.68	–	NG_056945	NG_060969	–	–	–
	*Arthroderma curreyi*	CBS 138.26	KT155805	AY176726	AJ315165	–	–	–
	*Ctenomyces serratus*	CBS 187.61	NR_144890	NG_058765	NG_062605	–	–	–
	*Eremascus albus*	CBS 975.69	MH859498	MH871279	FJ358348	–	–	–
	*Gymnascella littoralis*	CBS 454.73	NR_155105	NG_057810	NG_062769	–	–	–
	*Nannizziopsis vriesii*	CBS 407.71	NR_111874	NG_057976	NG_061166	–	–	–
	*Paranannizziopsis australasiensis*	UAMH 10439	KF477218	–	KF466866	–	–	–
*Mycocaliciomycetidae*								
*Mycocaliciales*	*Brunneocarpos banksiae*	CBS 141465	NR_147648	MH878228	–	–	–	–
	*Chaenothecopsis golubkovae*	Titov 6707 UPS	AY795859	AY795996	–	–	–	–
	*Chaenothecopsis sitchensis*	HT22	–	KF157988	KF157976	–	–	–
	*Phaeocalicium polyporaeum*	ZW-Geo60-Clark	AY789363	AY789362	AY789361	–	–	–
*Sclerococcomycetidae *								
*Sclerococcales*	*Rhopalophora clavispora*	CBS 637.73	NR_152542	KX537757	NG_061246	–	–	–
	*Sclerococcum ricasoliae*	A.F.Fla6b	MT153964	MT153993	–	–	–	–
	*Sclerococcum vrijmoediae*	NTOU 4002	KJ958534	KC692153	KC692152	–	–	–
*LECANOROMYCETES*								
*Lecanoromycetidae *								
*Caliciales*	*Calicium glaucellum*	Tibell 22,319 UPS	AY450569	AY453646	–	–	–	–
	*Calicium salicinum*	CBS 100898	–	KF157982	KF157970	–	–	–

A.F: Adam Flaku´s personal collection, Department of Lichenology, W. Szafer Institute of Botany, Poland; AFTOL-ID: Assembling the Fungal Tree of Life (AFTOL) project (www.lutzonilab.net/aftol); ARAN-Fungi: ARAN-Fungi Fungarium, the Basque Country, Spain; CBS: Westerdijk Fungal Biodiversity Institute, The Netherlands; CCF: Culture Collection of Fungi, Department of Botany, Charles University in Prague, Czech Republic; Clark: Clark University, USA; CMW: Forestry and Agricultural Biotechnology Institute, University of Pretoria, South Africa; DAOM: Canadian National Mycological Herbarium, Ottawa Research and Development Centre, Canada; Etayo: Javier Etayo´s personal collection, Spain; F: Personal number of Zdenek Palice, Institute of Botany, Academy of Sciences of the Czech Republic, HT: personal number of Hanna Tuovila, University of Helsinki, Finland; MUCL: Mycotheque de l’Universite Catholique de Louvain, Belgium; NBRC: Biological Resource Center, NITE, Japan; NFCCI: National Fungal Culture Collection of India, India, NRRL: Agricultural Research Service (ARS) Culture collection, Illinois, USA; NTOU: National Taiwan Ocean University, Taiwan; PREM: National Collection of Fungi, South Africa; UPS: Museum of Evolution, Sweden; UAMH 10439: Centre for Global Microfungal Biodiversity, University of Toronto, Canada. New species are shown in bold

### Phylogenetic analyses

Two reference sequence datasets were constructed for the phylogenetic analyses (see Table 1). The first was mostly based on the Prieto et al. (2021) dataset and included SSU, ITS and LSU used to determine the placement of our new species within *Eurotiomycetes* using a multigene phylogenetic analysis. The second phylogenetic analysis was constructed to study the relationships between our strains by calculating single gene trees based on ITS, *RPB2*, *MCM7* and *TEF1* and applying the Genealogical Concordance Phylogenetic Species Recognition (GCPSR) approach (Taylor et al. 2000).

Each dataset was aligned using the MAFFT v.7 online service selecting the L-INS-i algorithm (Katoh et al. 2005, 2019). Alignments were visualized, trimmed and edited, where necessary, using MEGA v.7.0.26 (Kumar et al. 2016). For the multigene analysis, alignments were concatenated in CLC Main Workbench. Maximum likelihood (ML) trees were constructed using the IQ-TREE Web server (Trifinopoulos et al. 2016) selecting the best-suited nucleotide substitution model (partitioning the data in the case of concatenated trees) according to the Bayesian information criterion (BIC) using ModelFinder (Kalyaanamoorthy et al. 2017) built into IQ-TREE. Statistical support was calculated using the Ultrafast Bootstrap analysis (Minh et al. 2013). Phylogenetic trees were visualized in FigTree v.1.3.1 (http://tree.bio.ed.ac.uk/software/figtree/) and edited using Affinity Designer v.1.10.5.1342 (Serif (Europe), Nottingham, UK). Aligned datasets and IQ-TREE outputs were uploaded to the University of Pretoria’s research data repository hosted on Figshare and can be accessed at https://doi.org/10.25403/UPresearchdata.21640298.

### Morphology

#### Spore producing structures on branch samples

The fungi were characterised from their natural habitat on *A. araucana* branches as well as growing on standardised growth media including MEA, ½PDA, WA and oatmeal agar (OMA). Microscopic slides were prepared from 28 d old colonies using 10% lactic acid as mountant. Structures collected directly from samples were first sectioned using a Leica CM1520 cryostat (Deer Park, TX) before a microscope specimen was prepared. Characters were observed and photographed using a Zeiss AXIO Zoom.V16 dissection and AXIO Imager.A2 compound microscopes both equipped with a Zeiss AxioCaM 512 colour camera driven by Zen Blue v.3.2 software (Carl Zeiss CMP, Göttingen, Germany). For dissection microscope images, extended depth of field analyses were performed using Helicon Focus v.7.5.4 (HeliconSoft, Kharkiv, Ukraine). Up to 50 measurements were made for each character where available, and boxplot graphs were constructed with measurements of each character to visualize data distribution. Photographic plates were prepared in Affinity Designer v.1.10.5.1342 (Serif (Europe) Ltd, Nottingham, UK). Attempts were made at the time of the study to obtain specimens for *R. humboldtensis* but these were unsuccessful. Morphological comparisons were thus based on the description published in Rikkinen et al. (2016).

#### Colony growth rate

Colony growth rates for the new species were compared by growing strains under standardised conditions on MEA, PDA and WA. A spore suspension was prepared for each strain by cutting 10–20 agar blocks from 14 d old MEA colonies and suspending these in 10% glycerol. Growth media were subsequently inoculated at three equidistant spots (~ 1 µL each) using a micropipette and allowed to dry for 10 min before plates were sealed with Parafilm® and incubated upside down. Plates were incubated at temperature gradient of 5 °C intervals from 5–35 °C for 21 d and colony diameters measured every 3–4 d. The colony area was measured from images taken on day 21 using ImageJ v.1.52 (Abràmoff et al. 2004; Guerrero et al. 2012) software with an adjusted scale. Plates that showed no growth at particular temperatures were subsequently incubated at 20 °C for two additional weeks to determine whether the original inoculum had remained viable. Descriptions, colour names and codes were based on Kornerup and Wanscher (1967).

## RESULTS

### Isolations and preliminary identification

Isolations made from surface disinfested *Araucaria araucana* tissue collected from the four surveyed areas resulted in 23 strains. Direct isolation of an additional 25 strains were made from five ascomata observed on samples collected in Conguillío National Park (Andes Mountain range) and Trongol Alto (Coastal range).

DNA extraction and ITS sequencing was performed for all strains originating from plant tissue (*n* = 23), and from single spore isolates from five fruiting bodies (*n* = 5). A preliminary phylogenetic analysis (Additional file 1: Fig. S1) resolved the strains into two closely related clades. The first clade contained 22 strains and included all those originating from ascospores, while the second contained six strains. When sequences for the strains in both clades were compared using a BLAST search against the NCBI nucleotide database, the closest hits were *Cr. blascoi* (GenBank MW999967; Identity = 543/611(89%), 39 gaps (6%)), followed by *Penicillium scabrosum* (GenBank MT995062; Identity = 507/617(82%), 35 gaps (5%)), *P. hirayamae* (GenBank MH858553; Identity = 502/612(82%), 28 gaps (4%)), *P. copticola *(GenBank MF326615; Identity = 427/509(84%), 16 gaps (3%)), and *P. terrigenum* (GenBank KT336534; Identity = 508/617(82%), 27 gaps (4%)). Six strains were selected from each of the two ITS clades and five additional gene regions sequenced for them to continue further analyses.

### Phylogenetic analyses

**Fig. 1:**
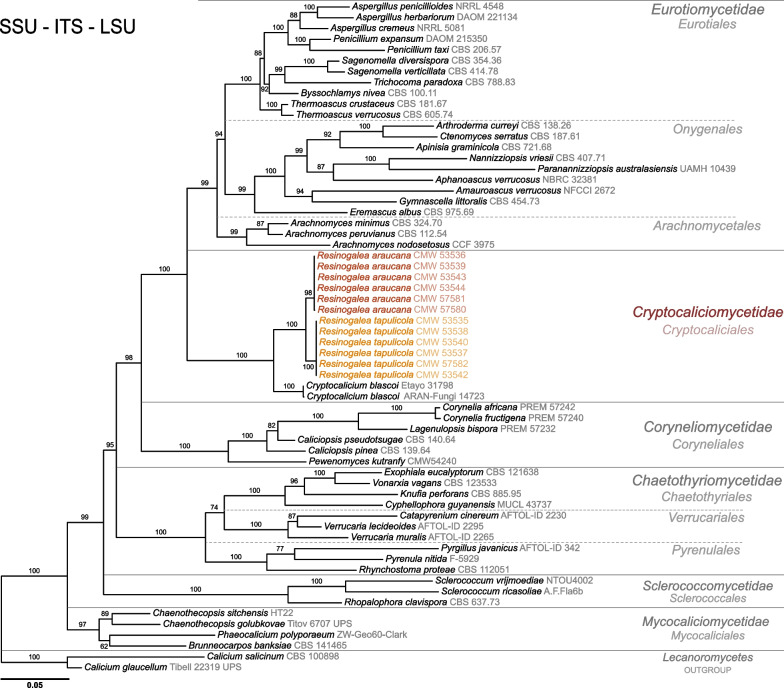
Maximum-likelihood phylogenetic tree for the concatenated nuc SSU, ITS, and nuc LSU gene sequences for the subclasses and part of the subsequent orders within *Eurotiomycetes*. Two species of *Calicium* (*Lecanoromycetes*) were chosen as the outgroups. Strains included in this study representing new species are shown in colour. Numbers on branches correspond to bootstrap values (n = 1,000)

A multigene phylogeny based on SSU, ITS and LSU was calculated to resolve the phylogenetic position of the 12 strains (six per clade resolved on preliminary analysis) in *Eurotiomycetes*. Alignments for SSU (52 taxa, 3911 bp), ITS (57 taxa, 835 bp) and LSU (61 taxa, 1024 bp) were concatenated resulting in a matrix containing 62 taxa that was 5770 bp long. The most suitable nucleotide substitution models according to the Bayesian information criterion (BIC) for SSU, ITS and LSU were TNe + I + G4, GTR + F + I + G4, and TN + F + I + G4 respectively. ML and BI analyses resolved strains, with high bootstrap support, into two clades in the subclass *Cryptocaliciomycetidae* with *Cr. blascoi* as its closest relative (Fig. 1).

**Fig. 2:**
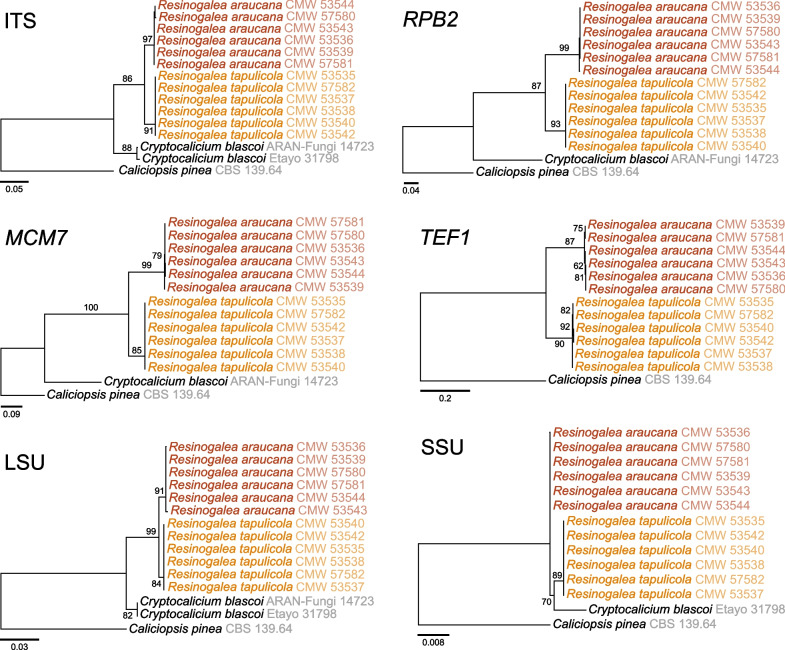
Maximum likelihood phylogenetic trees for the ITS, *RPB2*, *MCM7*, *TEF*, LSU and SSU gene sequences. *Caliciopsis pinea* (*Coryneliaceae*, *Coryneliales*) was chosen as the outgroup. Strains included in this study representing new species are shown in colour. Numbers on branches correspond to bootstrap values (n = 1,000)

Single gene phylogenies were subsequently calculated to assess the phylogenetic relationships between our strains by applying GCPSR (Fig. 2). For these, we included *Cr. blascoi* and the outgroup *Caliciopsis pinea*. Substitution models for the aligned ITS (16 taxa, 617 bp), *MCM7* (14 taxa, 618 bp), *RPB2* (14 taxa, 873 bp), and *TEF1* (13 taxa, 451 bp), datasets were, respectively, TNe + G4, K2P + I, TNe + I and TNe + G4. All gene regions resolved the strains in two concordant well-supported clades, and with the exception of SSU, all regions resolved them in a clade separate from *Cr. blascoi*. Similarities between isolates from the two clades containing our isolates were low; the SSU differed by three or more substitutions, the LSU differed by eight or more substitutions, the ITS differed by 20 or more substitutions, the *MCM7* differed by 99 or more substitutions, the *TEF1* differed by 102 or more substitutions, and the *RPB2* differed by 99 or more substitutions.

### Morphology

**Fig. 3:**
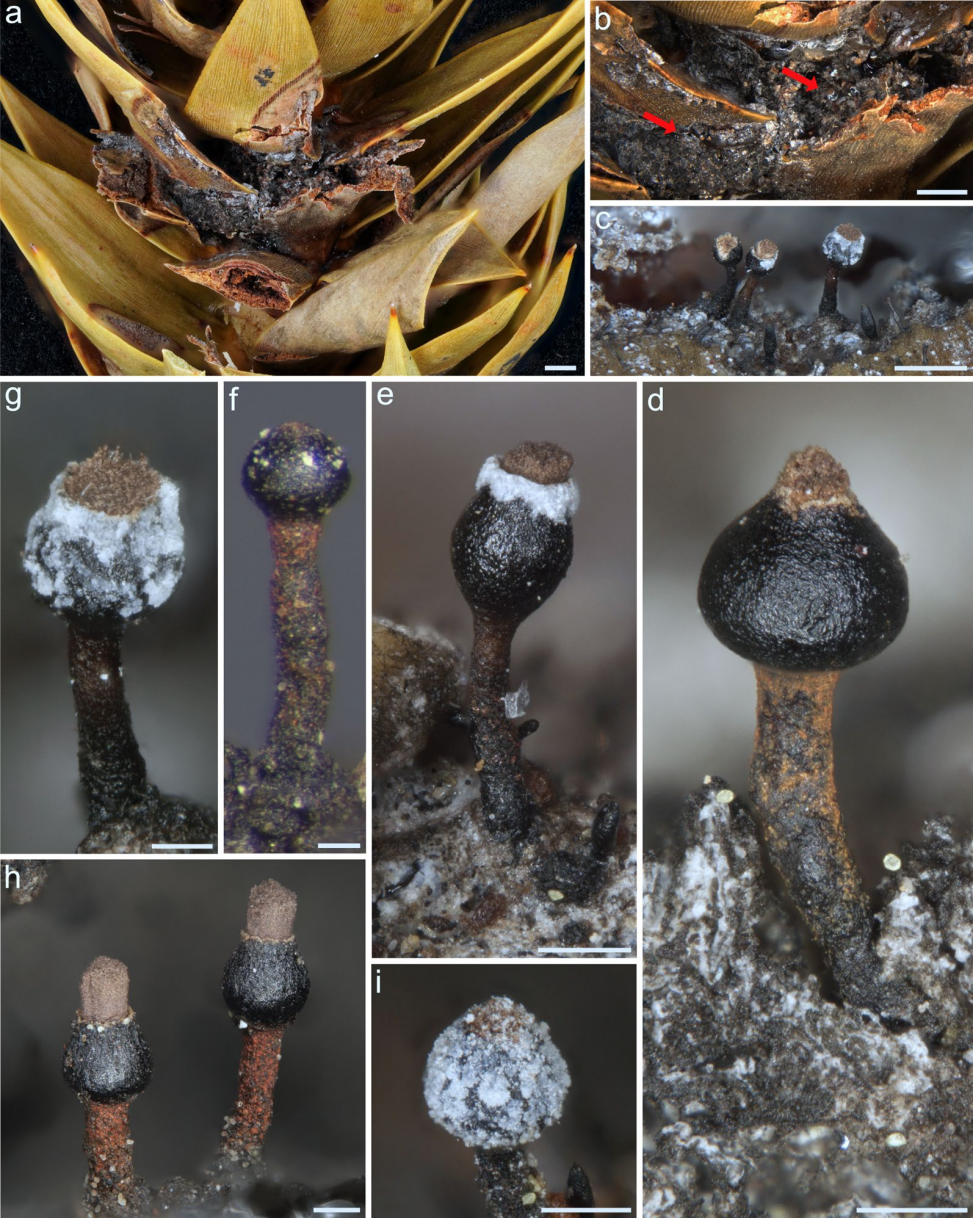
Ascomata from *Resinogalea araucana*. **a**, **b** Habit, dry resin patches on wounded branches of *Araucaria araucana*; **c**–**e** Ascomata emerging from and around resin patches; **f**, **g** Developing ascomata; **h** Mature ascomata with protruding columnar mazaedium; **i** Ascoma with capitulum covered with resin. Scale bars: **a**, **b** = 2000 µm; **c** = 500 µm; **d**, **e**, **h**, **i** = 200 µm; **f**, **g** = 100 µm

**Fig. 4:**
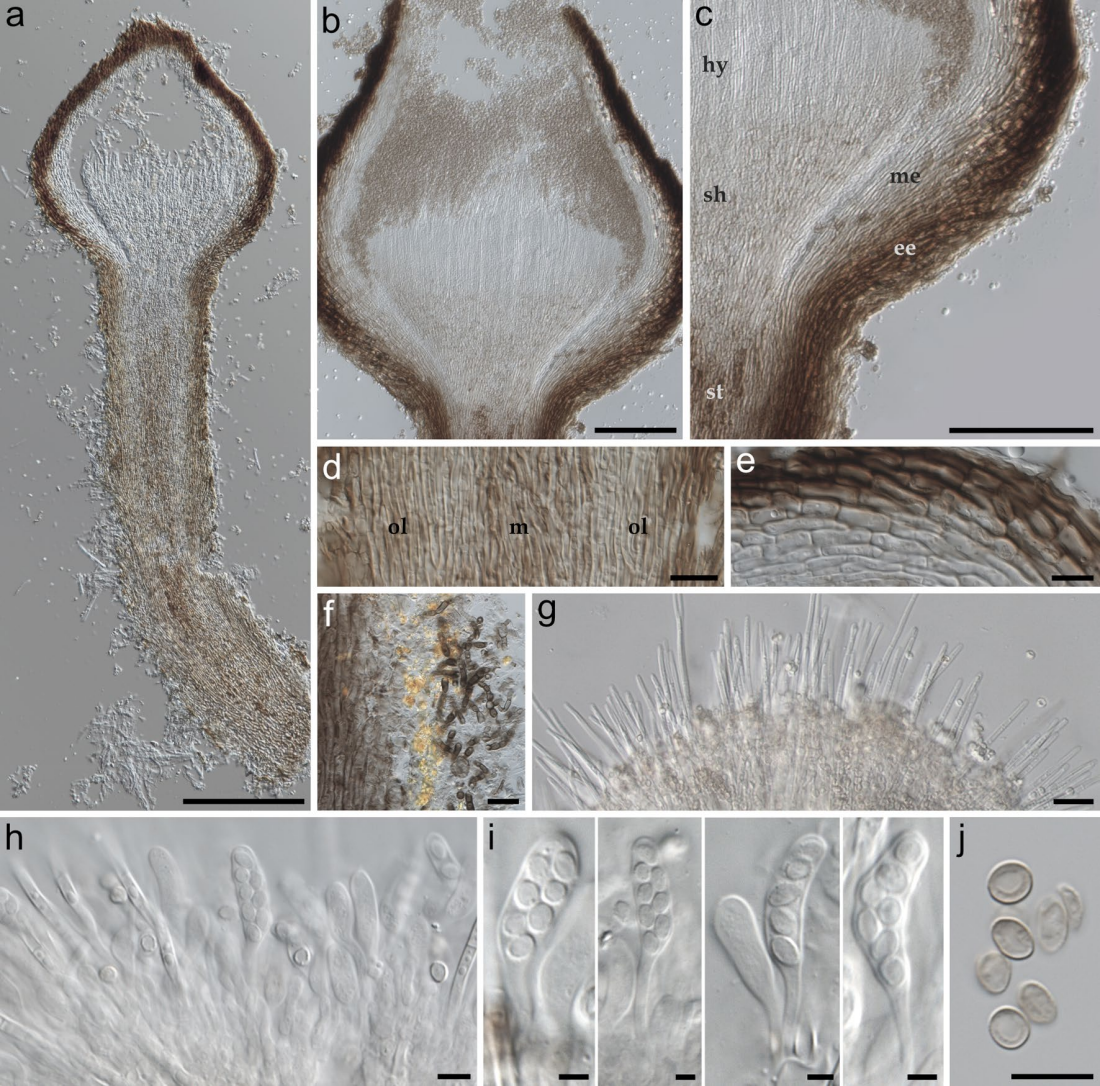
Micrograph of the ascomata of *Resinogalea araucana*. **a**–**c** Cross sections of ascomata; hy: hymeniun, sh: subhymenium, me: medullary excipulum, ee: ectal excipulum, st: stipe; **d**, **e** Textures, **d** stipe (m: medulla, ol: outer layers), **e** walls of the capitulum; **f** Mineral pruina and melanized dematiaceous hyphae at the outer layers of the base of the stipe; **g**, **h** Cross section of the hymenium containing paraphyses and developing asci; **i** Immature asci and ascospores; **j** Mature ascospores. Scale bars: **a** = 200 µm; **b**, **c** = 100 µm; **d**, **e** = 50 µm; **f**–**h** = 20 µm; **i**, **j** = 10 µm

Sexual sporing structures (ascomata) were observed only on branch samples, never in culture, and all strains obtained from them resided in a single clade based on DNA sequence comparisons. The ascomata on the samples emerged directly from resin or the plant tissue that surrounded it (Fig. 3). Ascomata of the species collected in this study were morphologically very similar to those described for *R. humboldtensis* (*Bruceomycetaceae*; Rikkinen et al. 2016) (Fig. 4, Table 2). They share additional features such as brownish orange mineral pruina that covered their stipes (Fig. 3, 4f), the production of mycelium that colonized the resin (Fig. 4f), a capitulum covered with resin (‘resin helmet’) (Fig. 3i), erythrocyte-shaped smooth to slightly roughened ascospores (Fig. 4j), and both occurred on resin patches of two *Araucaria* species (Fig. 3a, b). These characters were not observed in ascomata produced by *B. castoris*, the type of the *Bruceomycetaceae*. Additionally, *B. castoris* had distinctly larger dimensions for most structures (e.g. ascomata, ascospores) and distinctive characters such as the shape of the ascomata and ascospore ornamentation (Rikkinen 2003; Table 2).

**Table 2:** Morphological comparison of ascomata belonging to the novel *Resinogalea* found on *Araucaria araucana*, *Cryptocalicium blascoi*, *Resinogalea humboldtensis* and *Bruceomyces castoris*

Character	Dimension	*Resinogalea araucana* sp. nov	*Resinogalea humboldtensis*^1^	*Cryptocalicium blascoi*^2^	*Bruceomyces castoris*^3^
Habitat	Host	*Araucaria araucana*	*Araucaria humboldtensis*	*Juniperus* spp.	*Pseudotsuga*, *Abies, Acer*, *Alnus*
	Location	Chile	New Caledonia	Spain	USA
Ascomata	Height (µm)	(556) 766–1070 (1540)	650–1500	150–360	800–2500
Stipe	Length (µm)	(257) 364–598 (1069)	–	100–260	–
	Width (µm)	(70) 92–214 (275)	80–140	20–40	(75) 85–135 (200)
Hyphae on stipe	Width (µm)	2–3	(2.5) 3.0–4.5 (5.4)	(0.2–0.4) 1.2–1.6	–
Capitulum	Height (µm)	(121) 230–344 (500)	–	50–100	–
	Width (µm)	(204) 293–394 (467)	–	100–150	(170) 200–310 (430)
Mazaedium	Colour	Reddish brown	Reddish brown	Light ochre to greyish green	Pale greyish to reddish-brown
Ectal Excipulum	Cell width (µm)	3–6 (9)	–	3.5–5.5	–
Hymenium	Colour	Hyaline or slightly yellowish	Hyaline	Pale brown	Pale to medium brown, oil droplets
	Texture	Angularis–intricata	–	Angularis–globulosa	
Asci	Qualitative	Clavate, pedicellate, bitunicate, evanescent, 8–spored, often with biseriately arranged spores	Clavate, 8–spored, often with biseriately arranged spores	Clavate, bitunicate, initially thick–walled (wall 1 μm), then thin–walled, evanescent, 8–spored	Clavate, 8-spored, with relatively thick wall, not differentiated at the apex
	Length (total)	21–38 (57)	–	20–27	15–20
	Width (total)	4–6	6–9	5–7	7–9
	Sporiferous (L)	(15.5–)19–23(–30)	14–28	10–16	–
	Pedicel (L)	6–18.5(–32)	22–43	–	20–25
	Pedicel (W)	1.5–3	–	1 (diam.)	–
Ascospores	Shape	Broadly ellipsoidal varying to globose or ellipsoidal	Globose to broadly ellipsoidal, erythrocyte–like	Globose to subglobose, rarely ellipsoid	Ellipsoidal
	Colour	Pale brown	Pale brown	Pale brown	Pale brown
	Ornamentation	Smooth or slightly verrucose	Smooth, with very slight ornamentation visible in SEM	Smooth when viewed in a light microscope	Surface with longitudinal wrinkles visible under the light microscope
	Length (µm)	4–5.5 (6.5)	(3.0) 3.6 – 4.7 (5.8)	(3)3.3–4(4.7)	(6.0) 7.0–8.3 (9.8)
	Width (µm)	(3.5) 4–5.5	(2.7) 3.3 – 4.3 (4.6)	2.8–3.5 (4.0)	(4.0) 4.4–5.1 (5.8)
Paraphyses	Shape	Filiform, obtuse, non–branched	Filiform and non–branched	Cylindrical, obtuse	–
	Colour	Hyaline	Hyaline	Hyaline to very pale brown	–
	Septa (n)	(1) 2–4 (5)	–	2–4 septa	–
	Length (µm)	64–121(–172)	–	32–40	–
	Width (µm)	2–3	2.0–4.2	1.5–2	2.5–3.5

^1^Rikkinen et al. 2016; ^2^Prieto et al. 2020; ^3^Rikkinen 2003

*Cryptocalicium blascoi*, the closest phylogenetic relative to our species, shares broad morphological and niche features with our fungus and *R. humboldtensis* (Rikkinen et al. 2016; Prieto et al. 2021). These include growth on conifer resin, ascomata developing a definite capitulum, stipes covered with mineral pruina and producing columnar protruding mazaedia. However, its ascomata were considerably different when comparing dimensions (e.g. size of ascomata) and colour (mazaedium, mineral pruina; Table 2).

**Fig. 5:**
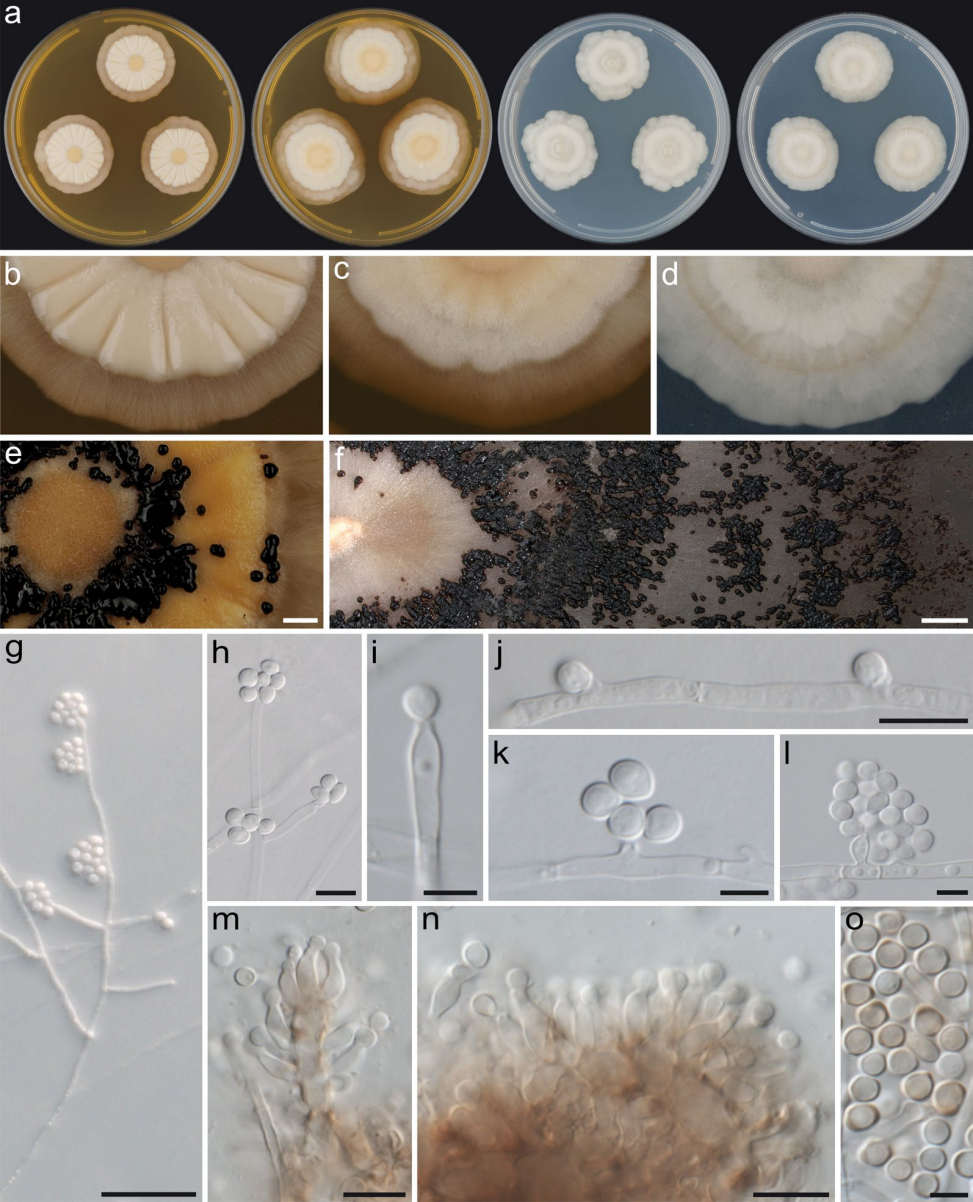
Culture morphology of *Resinogalea araucana*, isolate CMW 57580. **a** Colonies after 28 d in darkness, from left to right, MEA (20 °C), MEA (25 °C), PDA (20 °C), PDA (25 °C); **b**–**d** Colony textures on culture media: **b** MEA at 20 °C, **c** MEA at 25 °C, **d** PDA at 25 °C; **e**, **f** Sporodochia in 8-week or older cultures on MEA; **g** Conidiophores on mycelium; **h**–**l** Conidiogenous cells: terminal **h**–**i**, lateral denticles **j**–**k**, definite phialides (**l**); **m** Phialides on swollen and melanized hyphae; **n** Phialides emerging from pseudostromatic hyphae on sporodochia; **o** Conidia. Scale bars: **e**, **f** = 2 mm; **g** = 50 µm; **h**, **j**, **m**, **n** = 10 µm; **i**, **k**–**l**, **o** = 5 µm

**Fig. 6:**
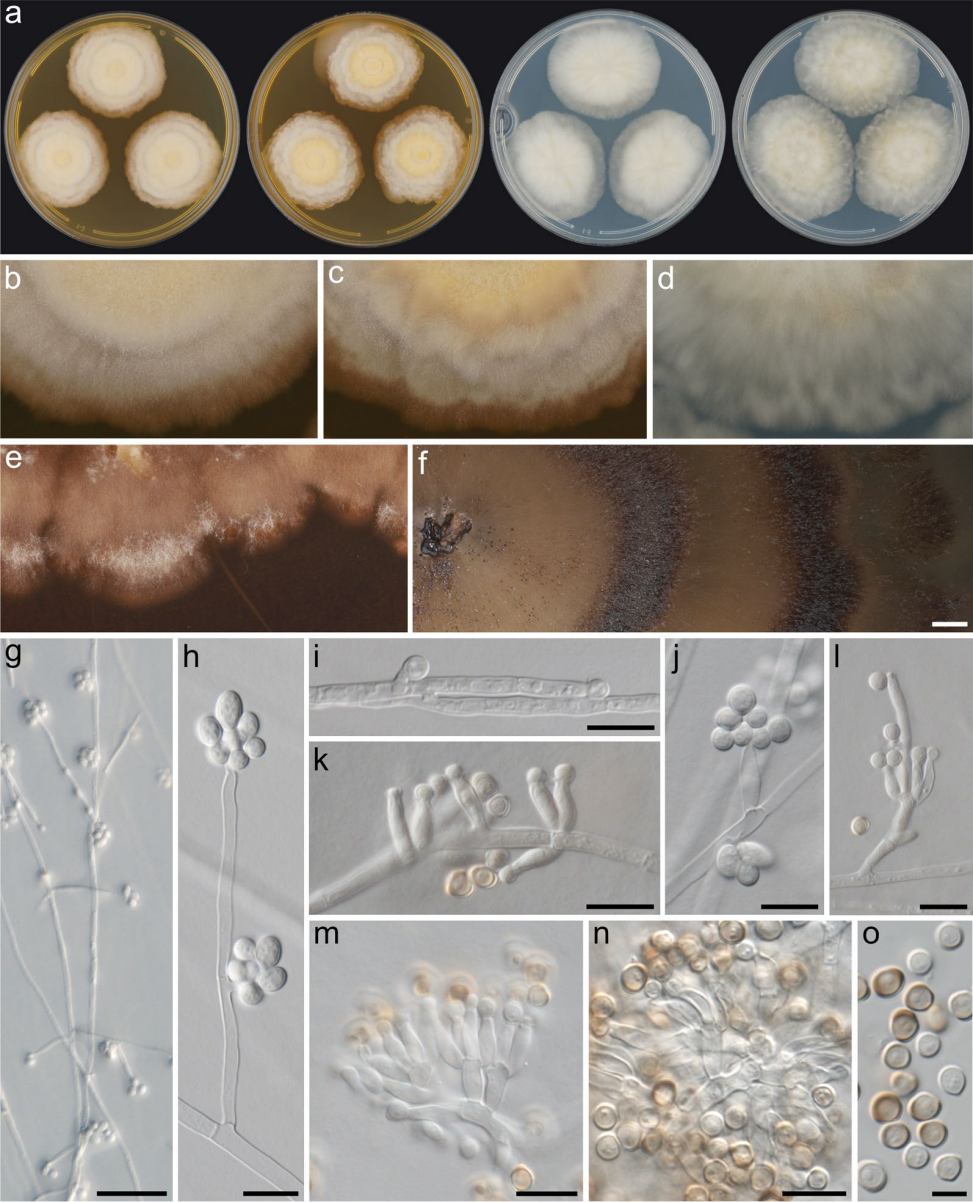
Culture morphology of *Resinogalea tapulicola*, isolate CMW 53537. **a** Colonies after 28 d in darkness, from left to right, MEA (20 °C), MEA (25 °C), PDA (20 °C), PDA (25 °C); **b**–**d** Colony textures on culture media: (b) MEA at 20 °C, **c** MEA at 25 °C, **d** PDA at 25 °C; **e**, **f** Morphological features on 8-week or older cultures on MEA: **e** staining of culture medium, **f** production of sporodochia; **g**–**h** Conidiophores on mycelium; **i**–**k** Conidiogenous cells: **i** terminal and lateral denticles, **j**, **k** definite phialides; **l**–**m** verticillate and richly branched conidiophores; **n** Phialides emerging from inflated and/or melanized hyphae on sporodochia; **o** Conidia. Scale bars: **f** = 2 mm; **g** = 50 µm; **h**–**n** = 10 µm; **o** = 5 µm

**Fig. 7:**
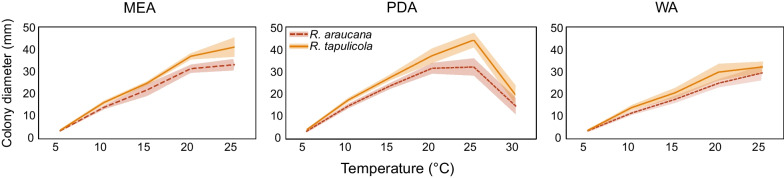
Culture growth after 28 days in darkness for two species of *Resinogalea* (six strains each) on three culture media (MEA, PDA and WA) and at different temperatures. Lines represent average and shaded areas represent standard deviation

In culture, all strains considered in this study had a broadly similar morphology when grown on any of the tested culture media, but they differed considerably from *Cr. blascoi* when morphology, colony growth rates and microscopic characters were compared (specified below). Furthermore, morphological characteristics, colony growth rate and optimal growth temperature data revealed minor but consistent differences between two species which we describe below (Figs. 5, 6, 7).

### Taxonomy

DNA sequence analyses and morphological comparisons showed that the fungi obtained from *Araucaria araucana* in this study belong in *Cryptocaliciomycetidae*. Furthermore, based on a polyphasic species concept they represented two distinct species. Although DNA sequence data and colony morphology are not available for the monotypic *R. humboldtensis*, this fungus shares an almost identical morphology, anatomy, and niche with the fungus found in the present study, sporulating on resin covering the branches of *A. araucana*. Additionally, the two species are more similar to each other than either is to *Bruceomyces castoris* (Table 2), the type species of *Bruceomycetaceae*, in which *R. humboldtensis* currently resides. Consequently, two new species in *Resinogalea* are described, and we reclassify the genus in the *Cryptocaliciomycetidae* alongside *Cryptocalicium*.

### *Resinogalea araucana* Balocchi & Visagie, sp. nov.

MycoBank: MB 846745.

*Etymology*: Latin, ‘*araucana*’*,* named after the type locality ‘Araucanía’ and the only known host, *Araucaria araucana*, on which this fungus colonizes resin patches.

*Diagnosis:* ITS barcode: OP508124. Alternative identification markers: LSU = OP508136, *MCM7* = OP524460, *RPB2* = OP524474, SSU = OP508112, *TEF1* = OP524486.

*Type:* **Chile**: *Araucanía (IX)*: Conguillío National Park sector Los Paraguas: -38.697836°, -71.817216°, from ascomata on branches of *A. araucana*, 02 May 2018, *F. Balocchi* (PREM = 63340, dried specimen in metabolically inactive state – holotype; CMW-IA: 55 = CMW 57581 = CBS 149622 = NCFB4 – ex-type cultures).

*Description: In nature* — *Ascomata* emerging from dry resin patches in branches of *A. araucana*, stipitate, capitate, (556–)766–1070(–1540) µm tall (excluding mazaedium); *stipe* cylindrical, subulate or rarely dumbbell-shaped, bent wholly or at the base, rarely straight, black, rugose, covered with a granular orange to reddish-brown mineral pruina, (257–)364–598(–1069) × (70–)92–214(–275) µm; *stipe medulla* composed of *textura intricata*-*porrecta*, brownish pigmentation; *stipe outermost layer* composed of 15–20 layers of *textura prismatica-porrecta*, long parallel hyphae, cylindrical, septate, slightly thick walled, hyaline inner layers becoming dematiaceous towards outer layers, hyphae 2–3 µm wide; *strongly melanized mycelium* emerging from the base of the stipe, hyphae darkly pigmentated and thick walled, (3–)6–9(–13.5) × 3–5(–7.5) µm; *capitulum* spherical when immature, ampulliform or urceolate when mature, (204–)293–394(–467) µm wide, (121–)230–344(–500) µm tall (not including mazaedium), black, rugose, sometimes covered with white to yellowish white resin; margins at the apical opening discrete, irregular, sometimes crenate-like, light brown; *mazaedium* well-developed in mature ascomata, reddish-brown, irregularly shaped or in some cases columnar, up to 290 µm in length; *ectal excipulum* poorly differentiated from the medullary excipulum, both combined consisting of 8–14 layers of *textura prismatica–porrecta*, hyphae periclinally arranged, darkly pigmented in the outer layers and becoming hyaline in the inner, (8–)11–17(–32) × 3–6(–9) µm; *subhymenium* hyaline or with very slight yellowish pigmentation, composed of *textura angularis-intricata*. *Asci* clavate, hyaline, pedicellate, bitunicate, evanescent, eight-spored, often with biseriately arranged spores, sporiferous part (15.5–)19–23(–30) × 4–6 µm, pedicel 6–18.5(–32) × 1.5–3 µm. *Ascospores* non-septate, hyaline when immature, becoming brownish with age, smooth to finely roughened, broadly ellipsoidal, some globose or ellipsoidal, some erythrocyte-like, 4–5.5(–6.5) × (3.5–)4–5.5 µm, (x̅ = 5.25 ± 0.6 × 4.34 ± 0.49), average length/width 1.22 ± 0.12 (*n* = 50). *Paraphyses* hyaline, filiform, obtuse, non-branched, 1–5 septate, 64–121(–172) × 2–3 µm. *Phialide-like* structures sporadically found inside the capitulum, potentially emerging from walls, hyaline, single-celled, subglobose, ellipsoidal or ampulliform, guttulate, (3–)4.5–7.5(–8.5) × 2–3(–4) µm, bearing single *conidia-like structures*, globose to obovoid, sometimes lobsided, hyaline when immature, becoming brownish with age, smooth or finely roughened, 4–6(–7) × 3.5–5.5 µm (x̅ = 5.54 ± 0.67 × 4.47 ± 0.59), average length/width = 1.25 ± 0.19 (*n* = 37).

*In culture — colony diameters*: (mm after 14 d (after 21 d)): MEA at 5 °C microcolonies (3–5), at 10 °C 6–9 (13–15), at 15 °C 13–21 (17–28), at 20 °C 19–26 (29–35), at 25 °C 18–24 (29–37); PDA at 5 °C microcolonies (3–4), at 10 °C 8–11 (13–17), at 15 °C 15–18 (21–26), at 20 °C 20–24 (28–35), at 25 °C 19–27 (27–40), at 30 °C 6–15 (8–21), at 35 °C no growth; WA at 5 °C microcolonies (2–5), at 10 °C 5–8 (10–13), at 15 °C 10–14 (15–20), at 20 °C 14–19 (22–27), at 25 °C 14–23 (25–36); OA at 20 °C 18–24 (30–36).

*Colony characters*: MEA 25 °C 21 d: Colonies velvety, concentric darkening or without pattern, umbonate, margins entire to slightly irregular, subsurface, *obverse* greyish yellow to dark yellow (4B4–C8), aerial mycelium yellowish white to orange white (4A2–5A2) at margins, pale to light yellow (4A3–5) centrally, *reverse* beige to blond (4C3–4) orange yellow to dark yellow (4B8–C7) centrally. MEA 20 °C 21 d: Colonies slimy, glabrous and sulcate, rarely dry and fluffy, umbonate, with full or slightly crenate margins, *obverse* greyish yellow to dark yellow (4B3–C8), aerial mycelium yellowish white to orange white (4A2–5A2) at margins, yellowish white to light yellow (4A2–5) centrally, *reverse* beige to blond (4C3–4) at margins, orange yellow to dark yellow (4B8–C7) centrally. MEA 20–25 °C 6 wks or older: Colonies slimy, glabrous, sulcate, irregular margins, producing sporodochia as mucilaginous spore masses on the surface, in concentric halos or with no pattern, dark brown to brownish black (6F4–H8). PDA 25 °C 21 d: Colonies velvety, glabrous, some cultures slimy only at centres, concentric or slightly petaloid pattern, umbonate, with full or slightly crenate margins, *obverse* yellowish grey to orange white (4B2–5A2), margins sunken in the medium, aerial mycelium greyish yellow to pale orange (4B4–5A3) at margins, becoming slightly darker or sometimes grey (4B1–D1) towards the centre, *reverse* yellowish white to yellowish grey (4A2–B2) at margins, pale yellow to olive (2D4–4A3) at centre, sometimes olive (2F7).

*Microscopic characters*: *Somatic hyphae* at colony periphery hyaline, smooth, thin-walled, branched, transversely septate, 1.5–4.5 µm diam, anastomosing and developing coils, becoming inflated and melanized with age. *Conidiomata* most commonly absent, sporodochial with age, composed of pseudostromatic thick-walled melanized hyphae, setae absent. *Conidiophores* reduced, unbranched, rarely single branched or stromatic. *Conidiogenous cells* monophialidic or undifferentiated, then acropleurogenous; *denticles* sometimes present, up to 3 µm in length; *terminal conidiogenous loci* 13.5–25 (–35) µm in length from septa; *phialides* ampulliform, curved or straight and tapering towards the apex, collarette distinct, (5.5–)7–10(–15) × 2.5–3(–4) µm. *Conidia* produced in slimy heads, hyaline, sometimes dematiaceous with age, aseptate, smooth walled, distinct hilum mostly present, globose to broadly ellipsoidal, larger conidia on PDA and WA typically ellipsoidal to ovoid, variable in size, from MEA (3–)3.5–4.5(–6.5) × 3–3.5(–4) µm (x̅ = 3.83 ± 0.46 × 3.41 ± 0.26), average length/width = 1.13 ± 0.17 (*n* = 228), from PDA 3.5–6(–8) × (3–)3.5–5 µm, (x̅ = 5.06 ± 1.11 × 3.82 ± 0.46), average length/width = 1.32 ± 0.26 (*n* = 56), from WA (3–)3.5–5(–6.5) × (2.5–)3–3.5 (–4.5) µm (x̅ = 4.39 ± 0.82 × 3.3 ± 0.38), average length/width = 1.34 ± 0.26 (*n* = 62).

*Habitat/host: Resinogalea araucana* produces ascomata on dry resin patches on damaged tissues in *A. araucana* branches. It was isolated directly from resin or plant tissues covered with resin. The type of damage inducing the exudated resin in which this fungus was found varied between samples, and included cankers caused by pathogenic fungi or insect feeding. This suggests that the presence of this fungus is merely associated with the presence of dry resin and has no role in causing resin to be produced. Strains originated from all collection sites in the Andes and Coastal range, suggesting that its distribution overlaps that of *A. araucana*.

*Material examined:* **Chile:** *Araucanía (IX)*: Conguillío National Park (sector Los Paraguas), -38.697836°, -71.817216°, from branches of *A. araucana*, 2 May 2018, *F. Balocchi* (PREM = 63340, dried specimen in metabolically inactive state; culture CMW 57580 = NCFB1); *ibid*, -38.697836°, -71.817216°, from branches of *A. araucana* (isolates from plant tissue covered in resin), 11 Dec. 2017, (culture CMW 53536 = AR023); *—* Villarrica National Park sector Puesco, -39.572706°, -71.499235°, branches of *A. araucana* (isolates from plant tissue covered in resin), 13 Dec. 2017, *F. Balocchi* (PREM = 63341, dried metabolically inactive culture; culture CMW 53539 = CMW-IA: 56 = CBS 149623 = AR140). *— Biobío (VIII)*, Ralco Natural Reserve, -37.962620°, -71.327679°, cankers on branches on *A. araucana* (isolates from plant tissue covered in resin), 27 Dec. 2017, *F. Balocchi* (culture CMW 53543 = AR224); *—* Nahuelbuta mountain range, Trongol Alto, -37.564893°, -73.205764°, branches of *A. araucana* (isolates from plant tissue covered in resin), 21 Dec. 2017, *F. Balocchi* (culture CMW 53544 = CMW-IA: 57 = CBS = 149,624 = AR290); — -37.553434°, -73.188438°, ascomata on branches of *A. araucana*, 5 Dec. 2019, *F. Balocchi* (PREM 63346, dried metabolically inactive specimen).

*Distinguishing characters*: See below.

### *Resinogalea tapulicola* Balocchi & Visagie, sp. nov.

MycoBank: MB 846746.

*Etymology:* Latin, ‘*tapulicola*’*,* named after the Mapudungun (indigenous Chilean and Argentinian Mapuche language) word ‘*tapül*’ meaning the leaves of a tree, and the Latin '-cola' meaning an inhabitant, inhabitant of leaves of a tree.

*Diagnosis:* ITS barcode: OP508126. Alternative identification markers: LSU = OP508138, *MCM7* = OP524462, *RPB2* = OP524476, SSU = OP508114, *TEF1* = OP524488.

*Type:* **Chile:** *Araucanía (IX)*: Conguillío National Park sector Los Paraguas, -38.697836°, -71.817216°, branches of *A. araucana* (isolates from plant tissue covered in resin), 11 Dec. 2017, *F. Balocchi* (PREM = 63343, dried specimen in metabolically inactive state – holotype; CMW-IA: 58 = CMW 53537 = CBS 149625 = AR073 – ex-type cultures).

*Description:* Only known in culture. *Colony diameters*: (mm after 14 d (after 21 d)): MEA at 5 °C microcolonies (3–4), at 10 °C 9–10 (15–17), at 15 °C 15–18 (24–26), at 20 °C 22–27 (33–39), at 25 °C 23–31 (33–47); PDA at 5 °C microcolonies (3–5), 10 °C 9–12 (16–19), 15 °C 17–20 (25–30), 20 °C 21–29 (32–44), at 25 °C 27–34 (39–49), 30 °C 11–19 (15–27), 35 °C no growth; WA at 5 °C microcolonies (3–5), at 10 °C 7–9 (10–16), at 15 °C 13–16 (17–24), at 20 °C 15–23 (22–34), at 25 °C 17–24 (29–36); OA at 20 °C 20–24 (29–37).

*Colony characters*: MEA 25 °C 21 d: Colonies velvety to tomentose, mycelium growth in a rosaceous and/or concentric pattern, umbonate, margins irregular, *obverse* dark yellow to greyish yellow (4B4–C8) at margins, aerial mycelium yellowish white to orange grey (4A2–5B2) at margins, pale to light yellow (4A3–5) centrally, *reverse* greyish yellow to blond (4B3–C4) at margins, becoming dark yellow to brownish yellow (4B8–5C7) centrally. MEA 20–25 °C 6 wks or older**:** slimy, glabrous, rarely velvety, umbonate, mycelium growing in a rosaceous and/or concentric pattern, margins irregular, soluble pigments golden brown to reddish brown (5D7–8E6), *obverse* orange grey to light brown (6B2–D4), aerial mycelium sometimes present, white to greyish red (3A1–7B4), *reverse* brownish orange to brown (5C3–F6), sporodochia appear as mucilaginous spore masses on the surface, in concentric halos or with no pattern, light brown to dark brown (6D6–F8). PDA 25 °C 21 d: slimy, glabrous to tomentose, mycelium growing in a rosaceous or stellate pattern, margins irregular, *obverse* edges sunken in the medium, yellowish grey (2B2–4B2), aerial mycelium white to pale yellow (3A1–3) at margins, becoming olive to dark grey (1F1–3E8) centrally, *reverse* pale grey to yellowish grey (3B1–3) at margins, becoming dull yellow to olive (3B4–E5) centrally, sometimes darker (3F7).

*Microscopic characters*: *Somatic hyphae* at colony periphery hyaline, smooth, thin-walled, branched, transversely septate, 1.5–4 µm diam, anastomosing, often developing coils, becoming inflated and melanized with age. *Conidiomata* most commonly absent, sporodochial with age, composed of interwoven thick-walled melanized hyphae, setae absent. *Conidiophores* reduced, unbranched, single branched, becoming monoverticillate to richly branched. *Conidiogenous cells* monophialidic or undifferentiated, then acropleurogenous; *denticles* sometimes present, up to 4.5 µm in length; *terminal conidiogenous loci* (10.5–)19–28(–52) µm in length from septa; *phialides* ampulliform, sometimes constricted at the middle, curved or straight and tapering towards the apex, with distinct collarette, (6–)8–12(–16) × (2–)2.5–3.5(–4) µm. *Conidia* produced in slimy heads, hyaline and thin-walled when young or on nutrient poor media, thick-walled and/or dematiaceous with age, aseptate, smooth walled, distinct hilum mostly present, on MEA globose to broadly ellipsoidal, rarely ellipsoidal, 3.5–4.5(–6) × 3–4.5 µm (x̅ = 4.28 ± 0.49 × 3.91 ± 0.32), average length/width = 1.09 ± 0.09 (*n* = 114), on PDA and WA subglobose to ellipsoidal, rarely globose, on PDA (3.5–)4–5(–6) × 3–4(–4.5) (x̅ = 4.66 ± 0.66 × 3.55 ± 0.38), average length/width = 1.32 ± 0.21 (*n* = 58), on WA (3–)4–5(–7.5) × (3–)3.5–4(–4.5) (x̅ = 4.31 ± 0.93 × 3.62 ± 0.38), average length/width = 1.19 ± 0.19 (*n* = 50).

*Habitat /host:* A sexual morph of this fungus has not been observed, and is only known from cultures isolated from plant tissues that had resin on them. Strains originate from collection sites in the Andes and Coastal range suggesting that its distribution overlaps that of *A. araucana*. Its occurrence on other tree species has not been explored and a broader geographical distribution may thus exist.

*Material examined:* **Chile:** *Araucanía (IX)*: Conguillío National Park sector Los Paraguas, -38.697836°, -71.817216°, branches of *A. araucana* (isolates from plant tissue covered in resin), 11 Dec. 2017, *F. Balocchi* (cultures CMW 53535 = AR011, CMW 53538 = AR074). *—* Villarrica National Park sector Puesco, -39.575582°, -71.493489°, branches of *A. araucana* (isolates from plant tissue covered in resin), 13 Dec. 2017, *F. Balocchi* (PREM = 63344, metabolically inactive culture; cultures CMW 53540 = CMW-IA:59 = CBS 149626 = AR149, CMW 53542 = AR155); *— Biobío (VIII)*, Nahuelbuta mountain range, Trongol Alto, -37.564893°, -73.205764°, branches of *A. araucana* (isolates from plant tissue covered in resin), 15 Jan. 2019, *F. Balocchi* (PREM = 63345, metabolically inactive culture; culture CMW 57582 = CMW-IA:60 = CBS 149627 = FB041).

*Distinguishing characters*: The general morphology of the ascomata produced by *R. araucana* closely resembles those of *R. humboldtensis,* and to a lesser extent *Cr. blascoi*. Ascomata were not observed for *R. tapulicola*. Comparing ascomata of *R. araucana* with those reported for *R. humboldtensis* (Rikkinen et al. 2016) showed those of *R. araucana* had a more robust and distinctively urn-shaped capitulum rather than spherical, which was only rarely fissured, not always covered by resin, and had a distinctive and well-defined apical opening often resulting in a cylindrical mazaedium. Stipes were wider ((70–)92–214(–275) µm *vs* 80–140 µm), and the mineral pruina covering it was orange to darker red as oppose to yellowish brown. Ascospores of *R. araucana* were on average slightly larger (4–5.5(–6.5) × (3.5–)4–5.5 µm *vs* (3–)3.6–4.7(–5.8) × (2.7–)3.3–4.3(–4.6) µm) and asci were thinner (4–6 µm *vs* 6–9 µm) with commonly shorter pedicels (6–18.5(–32) µm *vs* 22–43 µm). *Cryptocalicium blascoi* produces smaller ascomata (150–360 µm *vs* (556–)766–1070(–1540) µm), generally smaller microscopic features, and is characterized by a greenish mazaedium, contrasting with the reddish brown mazaedium of *R. araucana*.

Cultures of *R. araucana* and *R. tapulicola* resemble each other and could easily be confused. *Resinogalea tapulicola* grows faster than *R. araucana* (Fig. 7), has a drier texture (not slimy; Fig. 6a–d), mycelium commonly producing a slightly petaloid pattern and a reddish soluble pigment is produced on MEA (Fig. 6e). Microscopically, *R. tapulicola* produce more abundant coiled structures in the mycelium, has verticillated or richly branched conidiophores (Fig. 6l–m), and sporodochia produced from filamentous melanized hyphae (Fig. 6n) rather than pseudostromatic tissues as in *R. araucana* (Fig. 5n). *Resinogalea araucana* and *R. tapulicola* have several common morphological features in culture that serve to distinguish them from cultures of *Cr. blascoi* (Prieto et al. 2021). These include colony morphology (i.e. yellowish filamentous colonies) and production of sporodochial spore masses, and microscopic features such as conidia being produced from lateral denticles, well-developed phialides, and dematiaceous conidia.

## DISCUSSION

This study revealed two novel species of *Resinogalea* for which the names *R. araucana* and *R. tapulicola* are formally introduced. These unusual fungi were isolated from resinous areas on the branches of *A. araucana* in Chile. Ascomata were observed only in the case of *R. araucana*, while *R. tapulicola* is known only from culture. Based on broad morphological features of the *R. araucana* ascomata and the very specific niche in which these fungi occur, it is likely that their closest relative is *R. humboldtensis* (Rikkinen et al. 2016).

*Resinogalea humboldtensis* is a calicioid fungus also found on resin on branches of *A. humboldtensis* in New Caledonia. Our comparisons showed that *R. araucana* produces ascomata with similarities in shape and dimensions, as well as almost identical microscopic features, to those described for *R. humboldtensis* (Rikkinen et al. 2016; Table 2). Additionally, these species share some uncommon features, such as a stipe covered with a mineral layer, a capitulum covered with resin (‘resin helmet’), and the production of erythrocyte-shaped ascospores. Although all evidence suggests a close relationship between *R*. *araucana*, *R*. *tapulicola,* and *R. humboldtensis*, unfortunately the absence of DNA sequences and cultures for the latter makes it impossible to unequivocally confirm this relationship.

Rikkinen et al. (2016) described unsuccessful attempts to obtain DNA or fungal colonies from ascomata of *R. humboldtensis*. Based on this experience, we did not attempt DNA extractions directly from those of *R. araucana*. Instead, isolations onto culture media from these structures resulted in numerous fungal colonies. Ascospores from five ascomata germinated easily after 2–4 d on independent WA plates, and the identity of colonies (e.g. CMW 57581 and CMW 57580) originating from them was confirmed by sequencing. This difference in results between isolates from *R. araucana* and *R. humboldtensis* may be an outcome of different ages and/or preservation status of the specimens at their time of collection and/or processing. The erythrocyte-like shape of ascospores in *R. humboldtensis* observed by Rikkinen et al. (2016), rather than representing a true morphological feature, could be the result of spore collapse due to desiccation (Beckett et al. 1984). Although we found erythrocyte-like ascospores in *R. araucana* ascomata at high magnification, these were not common or consistently present as Rikkinen et al. (2016) reported for *R. humboldtensis*. The desiccation of ascospores could be the result of ascomata structures remaining for long periods on the tissues before degrading and/or exposure to dry and/or hot conditions, in both cases, also impairing the potential results of DNA extractions.

The results of our analyses using DNA sequence data generated from *R. tapulicola* and *R. araucana* showed that they reside in the subclass *Cryptocaliciomycetidae* (Prieto et al. 2021). This subclass, and subordinate taxonomic ranks, were described based on *Cr. blascoi*, and represent a discrete lineage of calicioid fungi within *Eurotiomycetes.* However, *R. humboldtensis* was described before the introduction of *Cr. blascoi*, and upon its description, based on morphological features, it was placed in the unresolved *Bruceomycetaceae* (*Ascomycota*, *incertae sedis*) alongside *B. castoris* (type species; Rikkinen 2003; Rikkinen et al. 2016). Based on our results that include DNA sequence data and morphological comparisons, we propose a reclassification of *Resinogalea* in *Cryptocaliciaceae* (*Cryptocaliciales*, *Cryptocaliciomycetidae*) with *Cryptocalicium*, the only other genus in the family. However, we consider *Bruceomyces* (*B. castoris*) to be morphologically sufficiently distinct from *Resinogalea* and *Cryptocalicium* to merit retention as the monotypic genus of *Bruceomycetaceae*. Furthermore, we emphasise that DNA sequence data will be needed to determine its relationship with other fungi.

In their study of *Cryptocalicium*, Prieto et al. (2021) described how major morphological features, such as ascomatal type, have progressively become obsolete to distinguish even between higher taxonomic groups. They were unable to assign *Cr. blascoi* to any taxonomic group in *Eurotiomycetes* and suggest that its specific taxonomic placement relied entirely on phylogenetic analyses. In earlier studies, Rikkinen et al. (2016) reported similar limitations when studying *R. humboldtensis*, where due to an absence of DNA sequences, that fungus remained in an unresolved taxonomic group (*Bruceomycetaceae*, *incertae sedis*). As a consequence, *R. humboldtensis* has remained taxonomically obscure and its relationship to the specimen we found on *A. araucana* was initially only suspected based on the hosts and substrate. This situation has been highlighted by numerous authors who note that a large number of old fungal names remain unsequenced (Brock et al. 2009; Dayarathne et al. 2016) and that conflict with current practices on species recognition which over rely on DNA sequence data (Dayarathne et al. 2016; Koukol and Delgado 2021). In consequence, species that lack associated DNA sequence data (e.g. absent in predominantly used databases such as GenBank) are easily omitted from studies (Aime et al. 2021; Koukol and Delgado 2021). Where possible, efforts need to be made to obtain sequences for the species that lack sequence data, e.g., sequencing old fungarium specimens (Larsson and Jacobsson 2004; Brock et al. 2009; Osmundson et al. 2013) and/or the collection and identification of new strains/specimens (Ariyawansa et al. 2014*, *Wood et al. 2016; *see Gonatobotrys simplex, *Crous et al. 2020). In our present and past studies on *A. araucana* (Balocchi et al. 2021, 2022a), this situation was found to be particularly relevant for fungi associated with this tree species and its close relatives in *Araucariaceae* (Balocchi et al. 2022b).

This study adds to several other species currently known to be associated only with *A. araucana* in Chile (Butin 1968, 1970a, 1970b, 1975, 1986), Grinbergs and Yarrow 1970, Butin 1975, 1986, Riess et al. 2016; Balocchi et al. 2021, 2022a). Similarly, a lichen-forming fungus (Boluda et al. 2015), several insect (Giganti and Dapoto 1990; Kuschel 2000; Mecke and Galileo 2004; Beéche 2017) and a mite species (Chetverikov et al. 2014) are specific associates of this ancient tree. Although limited in number, studies on the closely related *A. angustifolia* (Butin and Speer 1978; da Silva et al. 2015a, 2015b) and *A. humboldtensis* (Beimforde et al. 2017) have also found rare fungal species apparently only associated with them. *Araucaria* forests, and to a larger extent, *Araucariaceae* forests, have remained poorly studied in terms of microbial biodiversity (Balocchi et al. 2022b). Studies such as the present one provide a glimpse into the yet to be discovered diversity harboured by these ancient and iconic trees.

## CONCLUSION

This study combines morphological, ecological, and phylogenetic data to describe two novel *Resinogalea* species found in *A. araucana* in Chile. The genus *Resinogalea*, previously monotypic (*R. humboldtensis*), had no reference DNA sequence data available and had not been classified within the ascomycetes (*incertae sedis*). Morphological and ecological data supports our conclusions that the fungi collected in this study on *A. araucana* are most closely related to *R. humboldtensis*, and phylogenetic analyses showed that they reside in the recently described subclass *Cryptocaliciomycetidae*. The genus *Resinogalea* has consequently been re-classified in the *Cryptocaliciaceae* (*Cryptocaliciales*) together with the two new species *R. araucana* and *R. tapulicola* described here from *A. araucana* in Chile.

### Supplementary information

**Additional file 1.** Maximum likelihood tree for the ITS gene region for 28 isolates obtained from branches of *Araucaria araucana*.

## Data Availability

All sequence data generated in this study is available on GenBank (see Table 1). The datasets (alignments) generated and/or analysed during the current study are available in the Open Science Framework (OSF) repository, https://osf.io/d9zs7/?view_only=cd4e5c6e668347cfb98b72674c002fb7.

## Declarations

### Adherence to national and international regulations

Permissions for the collection and movement of samples from natural and protected areas were granted by the Chilean National Forest Corporation (CONAF) and Bioforest-Arauco SA. The importation of samples to South Africa was done under a permit held by the Forestry and Agricultural Biotechnology Institute (FABI), University of Pretoria, issued by the Department of Agriculture, Forestry and Fisheries of South Africa.

## References

[CR1] Abràmoff MD, Magalhães PJ, Ram SJ (2004). Image processing with ImageJ. Biophoton Int.

[CR2] Aime MC, Miller AN, Aoki T, Bensch K, Cai L, Crous PW, Hawksworth DL, Hyde KD, Kirk PM, Lücking R (2021). How to publish a new fungal species, or name, version 3.0. IMA Fungus.

[CR3] Ariyawansa HA, Hawksworth DL, Hyde KD, Jones E, Maharachchikumbura SS, Manamgoda DS, Thambugala KM, Udayanga D, Camporesi E, Daranagama A (2014). Epitypification and neotypification: guidelines with appropriate and inappropriate examples. Fungal Divers.

[CR4] Balocchi F, Marincowitz S, Wingfield MJ, Ahumada R, Barnes I (2022). Three new species of *Pewenomyces* (*Coryneliaceae*) from *Araucaria araucana* in Chile. Mycol Prog.

[CR5] Balocchi F, Wingfield MJ, Ahumada R, Barnes I (2021). *Pewenomyces kutranfy* gen nov. et sp. nov. causal agent of an important canker disease on *Araucaria araucana* in Chile. Plant Pathol.

[CR6] Balocchi F, Wingfield MJ, Paap T, Ahumada R, Barnes I (2022). Pathogens of the *Araucariaceae*: how much do we know?. Curr For Rep.

[CR7] Barnes I, Roux J, Wingfield MJ, Coetzee MP, Wingfield BD (2001). Characterization of *Seiridium* spp. associated with cypress canker based on ß-tubulin and histone sequences. Plant Dis.

[CR8] Beckett A, Read N, Porter R (1984). Variations in fungal spore dimensions in relation to preparatory techniques for light microscopy and scanning electron microscopy. J Microsc.

[CR9] Beéche MA (2017). *Yanara*, new genus of *Oecophoridae* (*Lepidoptera*) associated with *Araucaria araucana* (*Araucariaceae*) from southern Chile. Bol Cient Mus Hist.

[CR10] Beimforde C, Seyfullah LJ, Perrichot V, Schmidt K, Rikkinen J, Schmidt AR (2017). Resin exudation and resinicolous communities on *Araucaria humboldtensis* in New Caledonia. Arthropod Plant Interact.

[CR11] Boluda CG, Divakar PK, Hawksworth DL, Villagra J, Rico VJ (2015). Molecular studies reveal a new species of *Bryoria* in Chile. Lichenologist.

[CR12] Brock PM, Döring H, Bidartondo MI (2009). How to know unknown fungi: the role of a herbarium. New Phytol.

[CR13] Butin H (1968). A new species of *Ceratocystis* causing blue-stain in *Araucaria araucana*. Can J Bot.

[CR14] Butin H (1970). Zwei neue arten der gattung *Phaeocryptopus* naumov. J Phytopathol.

[CR15] Butin H (1970). Zwei neue *Caliciopsis*-arten auf chilenischen koniferen. J Phytopathol.

[CR16] Butin H (1975). Beitrag zur ascomyceten flora von Chile. Sydowia.

[CR17] Butin H (1986). *Rhizothyrium parasiticum* sp. nov. (Coelomycetes), ein blattparasit auf* Araucaria araucan*a (Mol.) C. Koch J Phytopathol.

[CR18] Butin H, Speer EO (1978). Über einige parasitische ascomyceten auf nadeln der Brasilianischen araukarie. Sydowia.

[CR19] Carbone I, Kohn LM (1999). A method for designing primer sets for speciation studies in filamentous ascomycetes. Mycologia.

[CR20] Carpintero DL, Dellapé PM (2006). *Pehuencoris*, a new genus of *Cardiastethini* (*Heteroptera*: *Anthocoridae*) from southern Argentina and Chile (Patagonia). Zool Sci.

[CR21] Chetverikov PE, Beaulieu F, Beliavskaia AY, Rautian MS, Sukhareva SI (2014). Redescription of an early-derivative mite, *Pentasetacus araucariae* (*Eriophyoidea*, *Phytoptidae*), and new hypotheses on the eriophyoid reproductive anatomy. Exp Appl Acarol.

[CR22] Crous P, Wingfield M, Schumacher R, Akulov A, Bulgakov T, Carnegie A, Jurjević Ž, Decock C, Denman S, Lombard L (2020). New and interesting fungi. Fungal Syst Evol.

[CR23] da Silva SS, Gusmão LFP, Castañeda-Ruiz RF (2015). Conidial fungi on *Araucaria angustifolia: Trichoconis foliicola* sp. nov. and two new records from Brazil. Mycotaxon.

[CR24] da Silva SS, Gusmão LFP, Castañeda-Ruiz RF (2015). *Cryptocoryneum parvulum*, a new species on *Araucaria angustifolia* (Brazilian pine). Mycotaxon.

[CR25] Dayarathne M, Boonmee S, Braun U, Crous PW, Daranagama DA, Dissanayake AJ, Ekanayaka H, Jayawardena R, Jones EBG, Maharachchikumbura SSNP, R.H., Phillips AJL, Stadler M, Thambugala KM, Wanasinghe DN, Zhao Q, Hyde KD, Jeewon R, (2016). Taxonomic utility of old names in current fungal classification and nomenclature: Conflicts, confusion & clarifications. Mycosphere.

[CR26] De Beer ZW, Duong T, Barnes I, Wingfield BD, Wingfield MJ (2014). Redefining *Ceratocystis* and allied genera. Stud Mycol.

[CR27] Duong TA, De Beer ZW, Wingfield BD, Wingfield MJ (2013). Characterization of the mating-type genes in *Leptographium procerum* and *Leptographium profanum*. Fungal Biol.

[CR28] Giganti H, Dapoto G (1990). Coleópteros de los bosques nativos del Departamento Aluminé (Neuquén-Argentina). Bosque.

[CR29] Grinbergs J, Yarrow D (1970). *Rhodotorula araucariae* sp. n. Antonie Van Leeuwenhoek.

[CR30] Guerrero NR, Quintero MAO, Naranjo JCP (2012). Determinación del área foliar en fotografías tomadas con una cámara web, un teléfono celular o una cámara semiprofesional. Rev Fac Nac Agron Medellin.

[CR31] Kalyaanamoorthy S, Minh BQ, Wong TK, von Haeseler A, Jermiin LS (2017). ModelFinder: fast model selection for accurate phylogenetic estimates. Nat Methods.

[CR32] Katoh K, Kuma K-i, Toh H, Miyata T (2005). MAFFT version 5: improvement in accuracy of multiple sequence alignment. Nucleic Acids Res.

[CR33] Katoh K, Rozewicki J, Yamada KD (2019). MAFFT online service: multiple sequence alignment, interactive sequence choice and visualization. Brief Bioinformatics.

[CR34] Kornerup A, Wanscher JH (1967). Methuen handbook of colour.

[CR35] Koukol O, Delgado G (2021). Why morphology matters: the negative consequences of hasty descriptions of putative novelties in asexual ascomycetes. IMA Fungus.

[CR36] Kumar S, Stecher G, Tamura K (2016). MEGA7: molecular evolutionary genetics analysis version 7.0 for bigger datasets. Mol Biol Evol.

[CR37] Kuschel G (2000). Curculionid (*Coleoptera*: *Curculionoidea*) fauna of *Araucaria araucana*. Rev Chil Entomol.

[CR38] Larsson E, Jacobsson S (2004). Controversy over *Hygrophorus cossus* settled using ITS sequence data from 200 year-old type material. Mycol Res.

[CR39] Liu YJ, Whelen S, Hall BD (1999). Phylogenetic relationships among ascomycetes: evidence from an RNA polymerse II subunit. Mol Biol Evol.

[CR40] Mecke R, Galileo MHM (2004). A review of the weevil fauna (*Coleoptera*, *Curculionoidea*) of *Araucaria angustifolia* (Bert.) O. Kuntze (*Araucariaceae*) in South Brazil. Rev Bras Zool.

[CR41] Minh BQ, Nguyen MAT, von Haeseler A (2013). Ultrafast approximation for phylogenetic bootstrap. Mol Biol Evol.

[CR42] O’Donnell K, Kistler HC, Cigelnik E, Ploetz RC (1998). Multiple evolutionary origins of the fungus causing Panama disease of banana: concordant evidence from nuclear and mitochondrial gene genealogies. Proc Natl Acad Sci.

[CR43] Osmundson TW, Robert VA, Schoch CL, Baker LJ, Smith A, Robich G, Mizzan L, Garbelotto MM (2013). Filling gaps in biodiversity knowledge for macrofungi: contributions and assessment of an herbarium collection DNA barcode sequencing project. PloS One.

[CR44] Prieto M, Etayo J, Olariaga I (2021). A new lineage of mazaediate fungi in the *Eurotiomycetes*: *Cryptocaliciomycetidae* subclass. nov., based on the new species *Cryptocalicium blascoi* and the revision of the ascoma evolution. Mycol Prog.

[CR45] Rehner SA, Samuels GJ (1995). Molecular systematics of the *Hypocreales*: a teleomorph gene phylogeny and the status of their anamorphs. Canad J Bot.

[CR46] Riess K, Schön ME, Lutz M, Butin H, Oberwinkler F, Garnica S (2016). On the evolutionary history of Uleiella chilensis, a smut fungus parasite of Araucaria araucana in South America: Uleiellales ord. nov. in Ustilaginomycetes. PloS One.

[CR47] Rikkinen J (2003). New resinicolous ascomycetes from beaver scars in western North America. Ann Bot Fenn.

[CR48] Rikkinen J, Beimforde C, Seyfullah LJ, Perrichot V, Schmidt K, Schmidt AR (2016). *Resinogalea humboldtensis* gen. et sp. nov., a new resinicolous fungus from New Caledonia, placed in *Bruceomycetaceae* fam. nova (*Ascomycota*). Ann Bot Fenn.

[CR49] Sung G-H, Sung J-M, Hywel-Jones NL, Spatafora JW (2007). A multi-gene phylogeny of *Clavicipitaceae* (*Ascomycota*, *Fungi*): identification of localized incongruence using a combinational bootstrap approach. Mol Phylogen Evol.

[CR50] Taylor JW, Jacobson DJ, Kroken S, Kasuga T, Geiser DM, Hibbett DS, Fisher MC (2000). Phylogenetic species recognition and species concepts in fungi. Fungal Genet Biol.

[CR51] Trifinopoulos J, Nguyen L-T, von Haeseler A, Minh BQ (2016). W-IQ-TREE: a fast online phylogenetic tool for maximum likelihood analysis. Nucleic Acids Res.

[CR52] Vilgalys R, Hester M (1990). Rapid genetic identification and mapping of enzymatically amplified ribosomal DNA from several *Cryptococcus* species. J Bacteriol.

[CR53] White TJ, Bruns T, Lee S, Taylor J, Innis MA, Gelfand DH, Sninsky JJ, White TJ (1990). Amplification and direct sequencing of fungal ribosomal RNA genes for phylogenetics. PCR protocols: a guide to methods and applications.

[CR54] Wood AR, Damm U, van der Linde EJ, Groenewald JZ, Cheewangkoon R, Crous PW (2016). Finding the missing link: Resolving the *Coryneliomycetidae* within *Eurotiomycetes*. Persoonia.

