# Supplementary material for: New Resinogalea species from Araucaria araucana resin in Chile and reclassification of the genus in the Cryptocaliciomycetidae
Source: IMA Fungus. 2023 Aug 18;14:16. doi: 10.1186/s43008-023-00122-9 (PMC10439659; doi:10.1186/s43008-023-00122-9)
Supplement: Supplementary file 1 — Additional file 1. Maximum likelihood tree for the ITS gene region for 28 isolates obtained from branches of Araucaria araucana. [file 43008_2023_122_MOESM1_ESM.pdf]

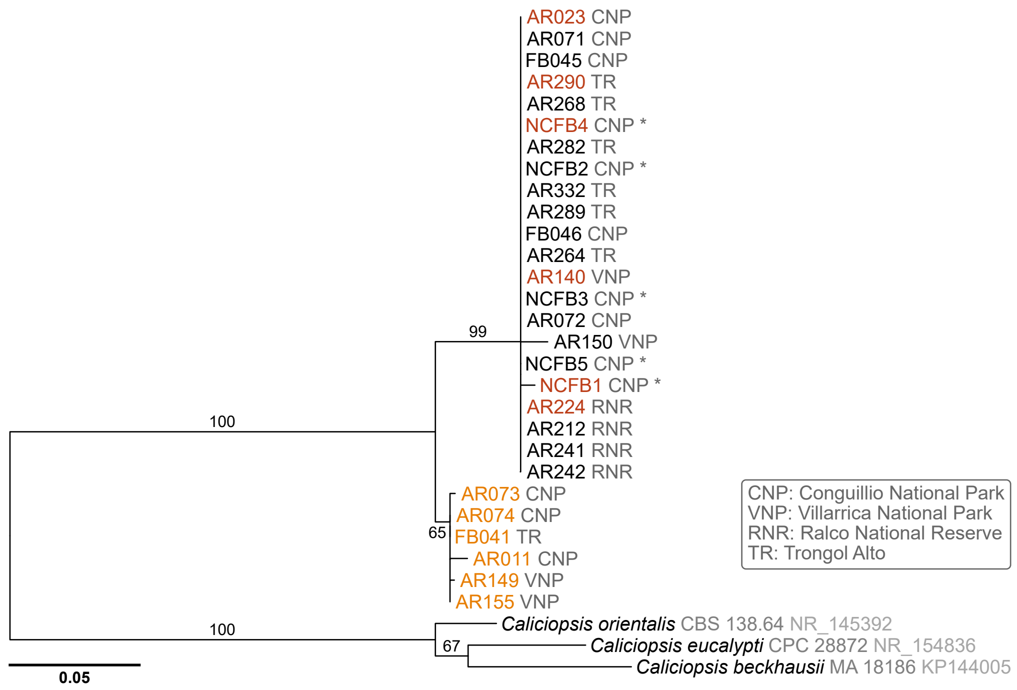

Supplementary Figure 1. Maximum likelihood tree for the ITS gene region for 28 isolates obtained from branches of *Araucaria araucana*. Isolated highlighted in colours were chosen for further phylogenetic analyses. *: Isolates obtained from single spore isolations from ascomata on the samples.
